# IL-1 Receptor Antagonist Antibodies in Idiopathic Recurrent Pericarditis

**DOI:** 10.1001/jamanetworkopen.2025.36691

**Published:** 2025-10-09

**Authors:** Maddalena Alessandra Wu, Christoph Kessel, Natalie Fadle, Evi Regitz, Sabrina Fuehner, Enrica Negro, Silvia Berra, Jochen Pfeifer, Elisa Ceriani, Bernhard Thurner, Karin Klingel, Alida Caforio, Massimo Imazio, Antonio Brucato, Lorenz Thurner

**Affiliations:** 1 Division of Internal Medicine, ASST Fatebenefratelli Sacco, Department of Biomedical and Clinical Sciences, University of Milan, Milan, Italy; 2 Department of Pediatric Rheumatology and Immunology, Unit for Translational Inflammation Research, University Children’s Hospital Muenster, Muenster, Germany; 3 José Carreras Center for Immuno and Gene Therapy and Department of Internal Medicine I, Saarland University, Homburg, Germany; 4 Department of Pediatrics, Klinikum Kempten, Kempten, Germany; 5 Cardiopathology, Institute for Pathology and Neuropathology, University Hospital Tübingen, Tübingen, Germany; 6 Department of Cardiac Thoracic Vascular Sciences and Public Health, University of Padua, Padua, Italy; 7 Department of Medicine, University of Udine, Udine, Italy; 8 Cardiothoracic Department, University Hospital Santa Maria della Misericordia, ASUFC, Udine, Italy

## Abstract

This cohort study investigates the prevalence and role of autoantibodies targeting the endogenous interleukin 1 (IL-1) receptor antagonist in patients with recurrent pericarditis with an inflammatory phenotype.

## Introduction

Idiopathic recurrent pericarditis is an autoinflammatory condition involving NLRP3 inflammasome activation and interleukin 1 (IL-1) release, leading to recurrent pericardial inflammation that is challenging to manage.^1^ Previous studies have also suggested an autoimmune contribution.^2^ In this study, we investigated the prevalence and role of autoantibodies targeting the endogenous IL-1 receptor antagonist (IL-1ra)^3^ in patients with recurrent pericarditis with an inflammatory phenotype.

## Methods

This prospective cohort PERIPLO study enrolled patients from 9 referral centers in Italy between June 1, 2023, and February 7, 2025. The study was approved by the Milano Area 1 Ethics Committee, and all participants provided written informed consent. The study followed the STROBE reporting guideline.

Plasma samples and clinical data were analyzed for proinflammatory autoantibodies, including anti–IL-1ra antibodies (IL-1ra-abs), and cytokine levels in a blinded fashion. When available, longitudinal samples from the same patient during active disease and remission were included for exploratory analysis. The eMethods in Supplement 1 provide additional information on the study design and procedures.

Data were analyzed using GraphPad Prism, version 10 for Windows or 10.4 for MacOS X (GraphPad Software) and SPSS, version 29.0.1 (IBM Corp). Individual markers were analyzed using Mann-Whitney *U *test. Datasets of more than 2 groups were analyzed using Kruskal-Wallis test followed by Dunn multiple comparison test. Significance was set at *P *< .05.

## Results

Among 142 participants (median [IQR] age, 49 [32.7-59.6] years; 80 female [56.3%] and 62 male [43.7%]), 49 (34.5%) were already receiving treatment with anakinra, a recombinant form of IL-1ra (Table). IL-1ra-abs were detected in 25 of 47 patients (53.2%) with active recurrent pericarditis and were not detected during remission (Table). Their presence frequently paralleled elevated C-reactive protein (CRP) levels at sampling (Figure, A). Patients positive for IL-1ra-abs exhibited peripheral IL-1ra depletion (Figure, B). While anakinra treatment increased circulating IL-1ra levels in both patients with active and inactive recurrent pericarditis, IL-1ra-ab seropositivity was not associated with anakinra exposure. In both the overall study cohort and patients not yet receiving anakinra at the time of sampling, those positive for IL-1ra-abs had elevated inflammatory markers, such as CRP and IL-6, vs those seronegative for IL-1ra-abs (Figure, C). IL-1ra antibodies formed immune complexes with their antigen and were associated with hyperphosphorylated IL-1ra (Figure, D). Antibodies to IL-18 binding protein and progranulin were either rare (progranulin) or not detectable (Table).

**Table.** PERIPLO Study Cohort

Characteristic	All participants (N = 142)^a^	Participants in acute phase of pericarditis (n = 47)	Participants in remission phase of pericarditis (n = 102)	*P* value
Age, median (IQR), y	49 (32.7-59.6)	52 (33.8-61.0)	47.6 (32.5-59.6)	.35
Sex, No. (%)				
Female	80 (56.3)	26 (55.3)	57 (55.9)	>.99
Male	62 (43.7)	21 (44.7)	45 (44.1)	
Recurrence rate, median (IQR), per y	1.3 (0.9-2.5)	1 (0.6-2.1)	1.4 (0.7-2.6)	.10
Hospital admissions, No. (%)	2.0 (1.0-3.0)	1.5 (1.0-3.0)	2.0 (1.0-3.0)	.44
Follow-up duration, median (IQR), y	2.0 (1.0-5.4)	1.1 (1.0-4.1)	2.4 (1.1-5.9)	.006
Maximum CRP, median (IQR), mg/L^b^	97.6 (30.9-186.2)	96.1 (45.0-217.0)	99.5 (27.6-170.0)	.25
CRP at sampling, median (IQR), mg/L^b^	4.0 (0.7-15.0)	27.1 (7.4-89.1)	1.9 (0.4-4.0)	<.001
IL-1ra-abs positive, No. (%)	25 (17.6)	25 (53.2)	0	<.001
IL-1ra plasma level, median (IQR), pg/mL	1457.0 (1225.3-2474.3)	1049.0 (95.0-1960.0)	1896.5 (1313.5-2642.8)	<.001
Neutrophil-to-lymphocyte ratio at first recurrence, median (IQR)	4.7 (2.7-6.8)	3.4 (2.0-5.5)	5.4 (3.3-7.0)	.049
Anakinra already taken, No. (%)	49 (34.5)	7 (14.9)	45 (44.1)	<.001
Anakinra started after first sampling, No. (%)	25 of 93 (26.9)	13 (32.5)	12 (21.1)	.24
IL-1ra-abs titer, median (IQR)	NA	1:400 (1:400 to 1:500)	NA	NA
IL-1ra-abs subclass	NA	24 IgG, 1 IgG and IgM	NA	NA
PGRN-abs	1	1	NA	NA
PGRN-ab titer	NA	1:400	NA	NA
PGRN-ab subclass	NA	IgG	NA	NA
IL-18bp-abs	ND	ND	ND	NA

Abbreviations: ab, antibody; IL-18bp, interleukin 18 binding protein; IL-1ra, interleukin 1 receptor antagonist; NA, not applicable; ND, not detectable; PGRN, progranulin.

^a^ Some participants contributed samples at multiple time points, resulting in a total of 149 samples.

^b^ To convert to milligrams per deciliter, divide by 10.

**Figure.**
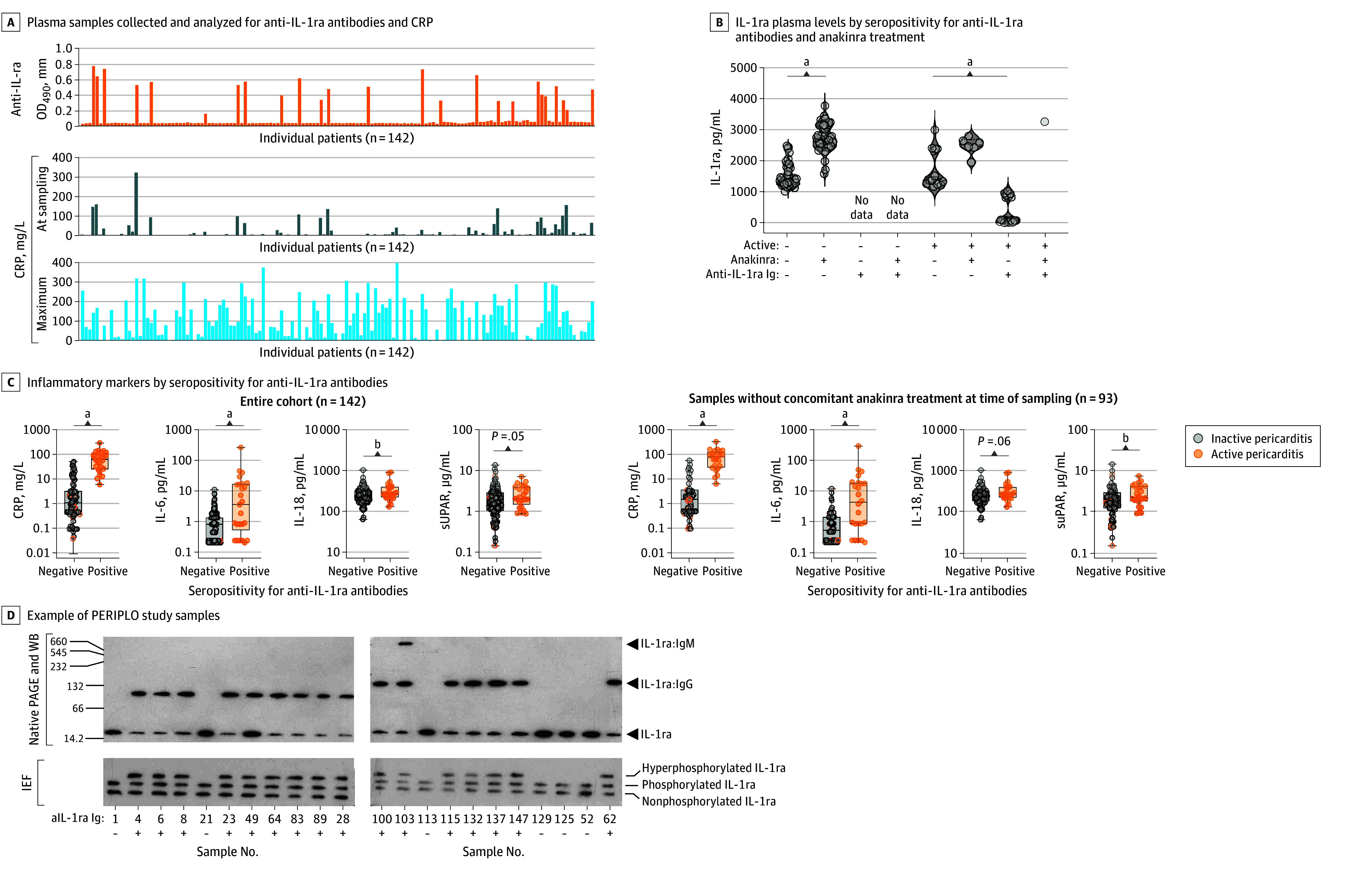
Anti–Interleukin 1 Receptor Antagonist (aIL-1ra) Antibodies in Idiopathic Recurrent Pericarditis, PERIPLO Study Cohort A, The PERIPLO study plasma samples were analyzed for aIL-1ra antibodies, C-reactive protein (CRP) at sampling, and maximum CRP levels. To convert CRP to milligrams per deciliter, divide by 10. B, IL-1ra plasma levels determined by commercial enzyme-linked immunosorbent assay and shown according to antibody status and anakinra treatment. Data were analyzed using Kruskal-Wallis test followed by Dunn multiple comparison test. C, Individual inflammatory markers quantified by multiplexed bead array assay and plotted according to seropositivity for aIL-1ra antibodies. Data were analyzed using Mann-Whitney *U* test. Boxes indicate the 25th percentile, median, and 75th percentile. Dots represent each sample. Whiskers show the minimum and maximum ranges. D, Representative plasma samples analyzed by native polyacrylamide gel electrophoresis (PAGE) or isoelectric focusing (IEF) followed by Western blot. Molecular weights are shown in kilodaltons. OD490, optical density at a wavelength of 490 nm; suPAR, soluble urokinase plasminogen activator receptor. ^a^*P* < .001. ^b^*P* < .05.

## Discussion

In this cohort study, IL-1ra-abs were exclusively detected in patients with active recurrent pericarditis and associated with inflammatory markers. A good correlation of seropositivity for IL-1Ra-abs with disease activity and molecular inflammatory markers, particularly in the context of heart inflammation as in ours and a previous study,^3^ may be related to the prominent role of IL-1 signaling in cardiac inflammation.^6^ In other inflammatory scenarios with multifaceted clinical presentation and evidence for IL-1ra-abs, such associations were not pronounced or evident.^4,5^

Here, IL-1ra-abs were associated with IL-1ra depletion and hyperphosphorylation, in line with prior reports suggesting a potential immunogenic role of posttranslational modifications.^3,4,5^ The transient nature and disappearance of IL-1ra-abs in longitudinal samples following inflammation resolution might support a possible association. In other investigations, patients’ IL-1ra-ab status did not correlate with other autoimmune features, such as antiheart antibodies and anti–intercalated disk antibodies,^2^ suggesting distinct pathogenic pathways. Importantly, anakinra treatment was not associated with autoantibody development.

Our findings were mainly limited by the small longitudinal sample size and the hypothesis-driven approach focusing on selected autoantibodies. Nonetheless, this study is the first to our knowledge to suggest transient IL-1ra autoimmunity as a feature of active recurrent pericarditis. These findings open potential avenues for biomarker development and improved disease monitoring.

## Notes

Supplement 1.
eMethods.

eReferences.

Supplement 2.
Data Sharing Statement

## References

[1] Cremer PC, Klein AL, Imazio M. Diagnosis, risk stratification, and treatment of pericarditis: a review. JAMA. 2024;332(13):1090-1100. doi:10.1001/jama.2024.12935 39235771

[2] Caforio AL, Brucato A, Doria A, . Anti-heart and anti-intercalated disk autoantibodies: evidence for autoimmunity in idiopathic recurrent acute pericarditis. Heart. 2010;96(10):779-784. doi:10.1136/hrt.2009.187138 20448129

[3] Thurner L, Kessel C, Fadle N, . IL-1RA antibodies in myocarditis after SARS-CoV-2 vaccination. N Engl J Med. 2022;387(16):1524-1527. doi:10.1056/NEJMc2205667 36130012 PMC9513854

[4] Hoffmann MC, Cavalli G, Fadle N, . Autoantibody-mediated depletion of IL-1RA in Still’s disease and potential impact of IL-1 targeting therapies. J Clin Immunol. 2024;44(2):45. doi:10.1007/s10875-023-01642-0 38231276 PMC10794369

[5] Pfeifer J, Thurner B, Kessel C, . Autoantibodies against interleukin-1 receptor antagonist in multisystem inflammatory syndrome in children: a multicentre, retrospective, cohort study. Lancet Rheumatol. 2022;4(5):e329-e337. doi:10.1016/S2665-9913(22)00064-9 35368387 PMC8963770

[6] Abbate A, Toldo S, Marchetti C, Kron J, Van Tassell BW, Dinarello CA. Interleukin-1 and the inflammasome as therapeutic targets in cardiovascular disease. Circ Res. 2020;126(9):1260-1280. doi:10.1161/CIRCRESAHA.120.315937 32324502 PMC8760628

