# Supplementary material for: IL-1 Receptor Antagonist Antibodies in Idiopathic Recurrent Pericarditis
Source: JAMA Netw Open. 2025 Oct 9;8(10):e2536691. doi: 10.1001/jamanetworkopen.2025.36691 (PMC12511996; doi:10.1001/jamanetworkopen.2025.36691)
Supplement: Supplement 1. — eMethods. eReferences. [file jamanetwopen-e2536691-s001.pdf]

Supplemental Online Content

Wu MA, Kessel C, Fadle N, et al. IL-1 receptor agonist antibodies in idiopathic recurrent pericarditis. *JAMA Netw Open*. 2025;8(10):e2536691.
doi:10.1001/jamanetworkopen.2025.36691

eMethods

eReferences

This supplemental material has been provided by the authors to give readers additional information about their work.

© 2025 Wu MA et al. *JAMA Network Open*.

eMethods

Study design and samples

The PERIPLO (PERicarditis: IL-1Ra antibodies and suPAR Levels Observational) Study is a prospective, multicenter, non-profit study (NCT05925790). The study was approved by the Ethics Committee and conducted in accordance with the Declaration of Helsinki. Patient samples were collected following written informed consent at the Division of Internal Medicine, ASST Fatebenefratelli Sacco, University of Milan, Italy. We enrolled patients with idiopathic RP, and a medical history indicative of a clear inflammatory phenotype with a history in all cases of acute attacks with elevated CRP during the attack. Recurrent pericarditis was defined according to the ESC guidelines(1), with the following exclusion criteria: age <18 years, connective tissue diseases, periodic fever syndromes, neoplastic or bacterial pericarditis. Blood samples were collected during either an active or inactive disease phase, as determined by clinical presentation and C-reactive protein (CRP) levels.

All proinflammatory autoantibody-related analyses of study samples were performed at Saarland University Hospital in Homburg (Germany). AHA and AIDA testing was conducted at the Cardioimmunology and Thrombosis Laboratory, Department of Cardiac Thoracic Vascular Sciences and Public Health, University of Padova. Cytokine and inflammatory marker levels in patients' plasma samples were assessed at the University Children's Hospital Münster (Germany). All study samples were analyzed in a blinded approach at all centers.

ELISA for anti-IL-1Ra, anti-PGRN and anti-IL-18bp antibodies

The ELISA for autoantibodies was performed as previously described(2-4). In short, antigens were obtained using the coding sequences of the *GRN* gene encoding PGRN, *IL1RN* and *IL18BP* and were recombinantly expressed with a C-terminal FLAG-tag in HEK293 cells under the control of a cytomegalovirus promoter (pSFI). Total cell extracts were prepared and bound to Nunc MaxiSorp plates (eBioscience, Frankfurt, Germany) precoated with murine anti-FLAG mAb at a dilution of 1:2500 (v/v; Sigma-Aldrich, Munich, Germany) at 4°C overnight. After blocking with 1.5% (w/v; 1h RT) gelatin in Tris-buffered saline (TBS) and washing steps with TBS with Triton X-100, the individual plasma samples were diluted 1:100 and added for 1h at RT. Following repeated washing steps and depending on the scientific question, different detection antibodies were added for 1h at RT (biotinylated goat anti-human heavy and light chain immunoglobulin G (IgG), 1:2500, Dianova, Hamburg, Germany; subclass-specific sheep anti-human IgG1, IgG2, IgG3 and IgG4, all 1:5000, Binding Site Group, Birmingham, UK; goat anti-human IgM, 1:2500, Dianova; goat anti-human IgA, 1:2500, Dianova). Following this step, corresponding biotinylated or peroxidase-labeled secondary antibodies were used for immunoassays carried out to detect IgG subclasses

© 2025 Wu MA et al. *JAMA Network Open*.

(anti-sheep IgG-POX, Merck; 1:5000, 1h RT) and IgA/M (biotinylated anti-goat IgG, Dianova; 1:2500, 1h RT). Assays were developed by addition of peroxidase-conjugated streptavidin (StreptPOX, 1:50.000, Roche Applied Science, Indianapolis, IN, USA; 10 min, RT) and OPD. As a cut-off for positivity, the average of the optical density (OD) of the negative samples plus three standard deviations was applied.

Analysis of IL-1Ra- or PGRN-immune complexes

For detection of immune complexed PGRN and IL-1Ra in patients' plasma (1:100, 15µl), samples were run on native gradient PAGE (4-20%, without reducing sample preparations and without SDS), followed by western blotting (tank transfer, 1h 100V) onto PVDF membranes. Membranes were blocked (o/n, 4°C in TBST/milk buffer (10% [v/v] milk in 10 mmol/L TrisHCl, pH7·5, 150 mmol/L NaCl, and 0·1% [v/v] Tween 20), washed and incubated (1h, RT) with either murine anti-hPGRN antibody (abcam ab169325, 1:2000) followed by anti-mouse/POX (Biorad, 170-6516, 1:3000), rabbit anti-hIL-1Ra antibody (antibodies-online #ABIN2856394, 1:2000) followed by anti-rabbit/POX (Biorad#170-6515, 1:3000), or biotinylated anti-human IgG antibody (Dianova, 109-066-097). All incubation steps were carried out for 1h at RT and followed by repeated washing steps in TBST. Finally, blots were incubated with Strep/Pox (Roche, 11089153001, 1:15000), washed and developed using ECL reagents.

Isoelectric focusing (IEF) and immunoblotting of IL-1Ra

For IEF of plasma IL-1Ra, plasma was pre-diluted 1:100 in 1xPBS and then mixed with IEF sample buffer at a ratio of 1:2. Samples were analyzed by IEF on a gel with a fixed pH gradient (pH 3–10) according to the manufacturer's instructions (Novex pH 3–10, Invitrogen, Germany, Karlsruhe) followed by immunoblotting. Western blotting and IEF of intracellular proteins were performed as already described. Immunoblotting of IEFs was performed by semi-dry blotting (1h) onto PVDF membranes. Blots were incubated with anti-IL-1Ra and anti-PGRN antibodies and developed as described above.

ELISA for IL-1Ra plasma levels

PGRN and IL-1Ra plasma levels were determined using commercially available ELISA kits (PGRN: AdipoGen, Incheon, South Korea; IL-1Ra: Invitrogen/ThermoFisher, BMS2080) according to the manufacturer's instructions.

IL-1β signaling reporter assay

For the IL-1β reporter assay, we used HEK-Blue™ IL-1β reporter cells (Invivogen, hkb-il1bv2), which respond to IL-1β and IL-1α signaling by induction of NF-κB/AP-1, leading to expression of a secreted embryonic alkaline phosphatase (SEAP) reporter. Anakinra at 40ng/ml, recombinant IL-

© 2025 Wu MA et al. *JAMA Network Open*.

1Ra at 40ng/ml (Biozol, PPT-AF-2000-01RA), anti-IL-1Ra antibody at 5 µg/ml (antibodies-online, ABIN2856394), recombinant SLP-antibody at 5µg/ml (abcam, ab191883), plasma diluted 1:20 from a COVID-19 patient with high-titer IL-1Ra-antibodies (Val 16) with and without recombinant IL-1Ra at 40ng/ml, and plasma diluted 1:20 from a COVID-19 patient without high-titer IL-1Ra antibodies (Val 44) with or without recombinant IL-1Ra at 40ng/ml were preincubated for 2h at room temperature. Subsequently, these samples were added together with either recombinant human IL-1β (Biozol, PPT-200-01B) or TNF (Biozol, PPT-300-01A) both at 2ng/ml in 100µl DMEM to 2x10^4 HEK-Blue™ IL-1β reporter cells per well and incubated overnight at 37°C. Thereafter, 180 µl of each supernatant was transferred, 20µl QUANTI-BlueTM (Invivogen, rep-qbs) was added, and SEAP activity was measured at OD of 650nm. Experiments were performed in triplicates.

Multiplexed bead array assay

Reagents for multiplexed quantification of IL-6, IL-18, and suPAR were purchased from R&D Systems (Minneapolis, OH, USA). Reagents and PERIPLO plasma samples were prepared according to the manufacturer's instructions (R&D Systems). Data acquisition and analysis was performed on a MAGPIX instrument (Merck Millipore, Darmstadt, Germany) using xPONENT v4.2 software (Luminex).

Anti-heart antibody (AHA) and anti-intercalated-disk antibody (AIDA) testing by standard indirect immunofluorescence

AHA and AIDA testing was conducted by standard indirect immunofluorescence at 1/10 dilution on 4 mm-thick unfixed fresh frozen cryostat sections of blood group O normal human atrium and skeletal muscle; AHA- and AIDA-positive sera were subsequently titrated to endpoint by double dilutions. Organ-specific and cross-reactive 1 AHA patterns were classified, as previously described(5).

Data analysis

Data were analyzed using using Graphpad Prism software (Version 10 for Windows or version 10.4 for Mac OS X, Graphpad Software, La Jolla, CA, USA) and SPSS Statistics version 29.0.1 (IBM Corp, Armonk, NY). Data of individual markers were analyzed by Mann-Whitney U t-test. Data sets comprising more than two groups were analyzed by multi-comparison analyses using Kruskal Wallis followed by Dunn's multiple comparison test. Overall, $P<0.05$ was considered statistically significant.

© 2025 Wu MA et al. *JAMA Network Open*.

eReferences

1. Adler Y, Charron P, Imazio M, Badano L, Baron-Esquivias G, Bogaert J, et al. 2015 ESC Guidelines for the diagnosis and management of pericardial diseases: The Task Force for the Diagnosis and Management of Pericardial Diseases of the European Society of Cardiology (ESC)Endorsed by: The European Association for Cardio-Thoracic Surgery (EACTS). Eur Heart J. 2015;36(42):2921-64.
2. Thurner L, Kessel C, Fadle N, Regitz E, Seidel F, Kindermann I, et al. IL-1RA Antibodies in Myocarditis after SARS-CoV-2 Vaccination. N Engl J Med. 2022;387(16):1524-7.
3. Hoffmann MC, Cavalli G, Fadle N, Cantoni E, Regitz E, Fleser O, et al. Autoantibody-Mediated Depletion of IL-1RA in Still's Disease and Potential Impact of IL-1 Targeting Therapies. J Clin Immunol. 2024;44(2):45.
4. Pfeifer J, Thurner B, Kessel C, Fadle N, Kheiroddin P, Regitz E, et al. Autoantibodies against interleukin-1 receptor antagonist in multisystem inflammatory syndrome in children: a multicentre, retrospective, cohort study. Lancet Rheumatol. 2022;4(5):e329-e37.
5. Caforio AL, Brucato A, Doria A, Brambilla G, Angelini A, Ghirardello A, et al. Anti-heart and anti-intercalated disk autoantibodies: evidence for autoimmunity in idiopathic recurrent acute pericarditis. Heart. 2010;96(10):779-84.

© 2025 Wu MA et al. *JAMA Network Open*.
