# Supplementary material for: IL-1 Receptor Antagonist Antibodies in Idiopathic Recurrent Pericarditis
Source: JAMA Netw Open. 2025 Oct 9;8(10):e2536691. doi: 10.1001/jamanetworkopen.2025.36691 (PMC12511996; doi:10.1001/jamanetworkopen.2025.36691)
Supplement: Supplement 2. — Data Sharing Statement [file jamanetwopen-e2536691-s002.pdf]

Data Sharing Statement

Data
Data available: No

Additional Information
Explanation for why data not available: Anonymized study data will be made avalibale by the corresponding author upon reasonable request
